# P-1424. Effectiveness of Live Attenuated and Inactivated Influenza Vaccines in Children: Interim data from the 2024/25 Influenza Season

**DOI:** 10.1093/ofid/ofaf695.1611

**Published:** 2026-01-11

**Authors:** Allyn Bandell, Wilhelmine Meeraus, Oliver Dibben, Fungwe Jah

**Affiliations:** AstraZeneca Biopharmaceuticals (Medical), Castle Rock, CO; AstraZeneca, Cambridge, England, United Kingdom; AstraZeneca, Cambridge, England, United Kingdom; AstraZeneca, Cambridge, England, United Kingdom

## Abstract

**Background:**

Annual influenza vaccination with live attenuated influenza vaccine (LAIV) or inactivated influenza vaccine (IIV) is recommended for children in several countries; it is the most effective way to protect children from influenza and minimize the risk of spreading the virus to family members and the community. Here we present vaccine effectiveness (VE) data for LAIV and IIV in children, gathered globally for the 2024/25 influenza season.

**Figure:**
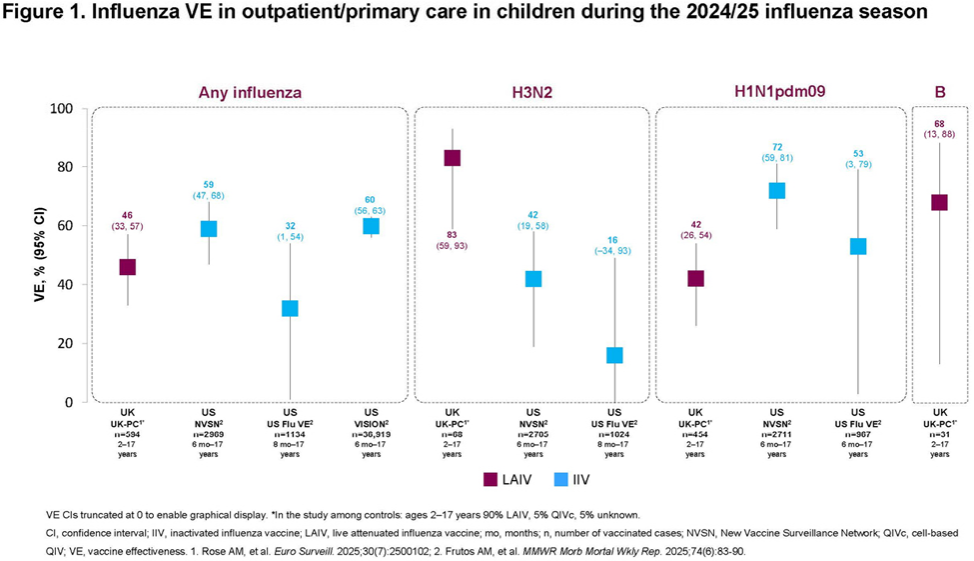

**Figure:**
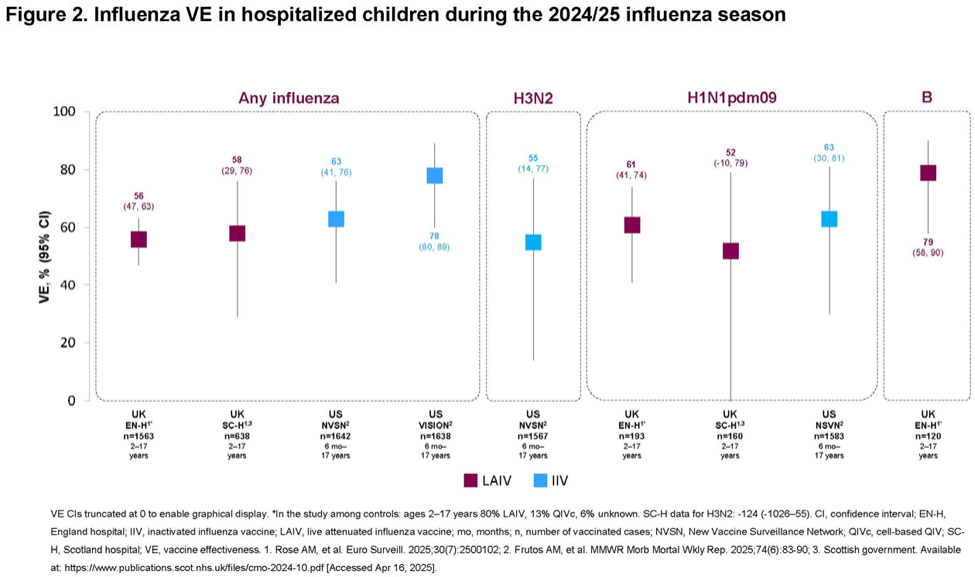

**Methods:**

Interim VE studies of LAIV and IIV in children during the 2024/25 influenza season were sourced from published data (to March 2025). Identified studies, conducted in the United Kingdom (UK) and the United States (US), provided VE data for any influenza infection (all strains), categorized by strain type (including A/H3N2, A/H1N1pdm09, and influenza B) and by vaccination setting (primary/outpatient care and hospital). Studies conducted in regions and countries such as Denmark and the EU without universal influenza vaccination recommendations in children, were excluded.

**Results:**

In a UK study, one Department of Defense study and three Center for Disease Control and Prevention (CDC)–affiliated US VE networks reporting on influenza infections (all strains) in children (UK: 2–17 years; US 0–17 years), interim VE estimates for LAIV were 46% to 58% in the UK, and for IIV were 19% to 78% in the US. When analyzed by vaccination setting where available, VE against any influenza, A/H3N2, A/H1N1pdm09 and influenza B ranged from 42% to 83% for LAIV and 16% to 72% for IIV in children in primary/outpatient care (Figure 1). For hospitalized children, VE against any influenza, A/H3N2, A/H1N1pdm09 and influenza B ranged from 52% to 79% for LAIV and 55% to 78% for IIV (Figure 2). Where available, VE data against any influenza, A/H3N2, A/H1N1pdm09 and influenza B were comparable for both LAIV and IIV children in primary/outpatient care and hospitalized children.

**Conclusion:**

Interim VE for the 2024/25 influenza season indicate that LAIV and IIV provided comparable and moderate protection for children against influenza. These VE data were consistent across outpatient/primary care and hospitals. Annual influenza vaccination with LAIV or IIV remains the most effective way to protect against influenza in multiple settings.

**Disclosures:**

Allyn Bandell, PharmD, AstraZeneca: Employee|AstraZeneca: Stocks/Bonds (Public Company) Wilhelmine Meeraus, PhD, AstraZeneca: Employee|AstraZeneca: Stocks/Bonds (Public Company) Oliver Dibben, PhD, AstraZeneca: Employee|AstraZeneca: Stocks/Bonds (Public Company) Fungwe Jah, MD, AstraZeneca: Employee|AstraZeneca: Stocks/Bonds (Public Company)

